# Guiding Pay-As-You-Live Health Insurance Models Toward Responsible Innovation in Health

**DOI:** 10.2196/19586

**Published:** 2020-08-21

**Authors:** Hassane Alami, Lysanne Rivard, Robson Rocha de Oliveira, Pascale Lehoux, Stéphanie Bernadette Mafalda Cadeddu, Mathilde Savoldelli, Mohamed Ali Ag Ahmed, Jean-Paul Fortin

**Affiliations:** 1 Center for Public Health Research Université de Montréal Montreal, QC Canada; 2 Department of Health Management, Evaluation, and Policy Université de Montréal Montreal, QC Canada; 3 Hospital Research Centre Université de Montréal Montreal, QC Canada; 4 Faculty of Law Université de Montréal Montreal, QC Canada; 5 School for Advanced Studies in Public Health Rennes France; 6 Research Chair on Chronic Diseases in Primary Care Université de Sherbrooke Chicoutimi, QC Canada; 7 Research Center on Healthcare and Services in Primary Care Université Laval Quebec, QC Canada; 8 Department of Social and Preventive Medicine Faculty of Medicine Université Laval Quebec, QC Canada

**Keywords:** digital health, client, Pay-As-You-Live, quantified-self, social contract, health insurance, responsible innovation in health, health system, health data, health inequity, health determinants

## Abstract

While the transition toward digitalized health care and service delivery challenges many publicly and privately funded health systems, patients are already producing a phenomenal amount of data on their health and lifestyle through their personal use of mobile technologies. To extract value from such user-generated data, a new insurance model is emerging called Pay-As-You-Live (PAYL). This model differs from other insurance models by offering to support clients in the management of their health in a more interactive yet directive manner. Despite significant promises for clients, there are critical issues that remain unaddressed, especially as PAYL models can significantly disrupt current collective insurance models and question the social contract in so-called universal and public health systems. In this paper, we discuss the following issues of concern: the quantification of health-related behavior, the burden of proof of compliance, client data privacy, and the potential threat to health insurance models based on risk mutualization. We explore how more responsible health insurance models in the digital health era could be developed, particularly by drawing from the Responsible Innovation in Health framework.

## Introduction

As health systems struggle to successfully implement a digital transition in care and service delivery [1], the ubiquity of mobile technologies combined with the emergence of the “quantified-self” movement has already generated a phenomenal amount of data on the health and lifestyle of individuals [2,3].

### Pay-As-You-Live: When Digital Health Technologies Influence New Insurance Models

Because of important financial incentives in the health sector, generated in part by certain social and health policies [4], health insurers can now create a new insurance model called Pay-As-You-Live (PAYL), which extracts value from user-generated data [5].

Partly driven by the success of the quantified-self movement [5], PAYL is a variant of the Pay-As-You-Drive care insurance model, which relies upon a GPS device (embedded in the car or a mobile application) to track client driving behavior to inform insurer decisions regarding increases or decreases in insurance premiums [6]. The insurer sends comments, information, and suggestions in real-time (via SMS text messaging or email) to inform the client about the appropriate driving behavior to adopt [5]. In this way, the insurer identifies the risks and leaves it up to the insured to decide, via the quality of their driving, the amount they will pay for the insurance.

As an interactive form of insurance, the principle of the PAYL model is to support clients in the management of their health. With real-time feedback, many insurers offer clients mobile applications and sensors to motivate them to adopt healthy behaviors and good lifestyle habits [5,7]. When using these applications and sensors intended to collect data, clients must share a multitude of health data with the insurer (for instance, weight, heart rate, eating habits, calorie intake, sleeping habits, places frequented, blood pressure, and clinical history) to track progress and judge compliance with the insurer’s recommendations. Clients who have achieved the defined objectives or have demonstrated a positive evolution over time are rewarded financially, especially in the form of bonuses (eg, capital of points) or reductions in the price of their insurance contract. As such, PAYL insurance models aim to reward clients who adopt healthy behaviors as much as they “punish” those who seem to choose to do otherwise.

Despite significant promises for clients, there are, in fact, critical issues that remain unaddressed, especially as PAYL models have the potential to significantly disrupt current insurance models and disproportionally impact more vulnerable segments of the population. In this paper, we discuss the following issues of concern: the quantification of health-related behavior, the burden of proof of compliance, client data privacy, and the potential threat to health insurance models based on risk mutualization. We then explore how more responsible health insurance models in the digital health era could be developed.

### Challenges to Quantifying Complex Individual Behaviors

The quantification of behavior derives from the assumption that life can be converted into digital data, or that quantitative measures of different activities and behaviors are constitutive of the person [8]. The reduction of individuals to a set of standardized measurements and quantifications poses two major problems.

First, there is a risk of omitting the complexity that surrounds individual and collective behaviors, particularly by underestimating the impact of systemic social determinants of health [3,9]. For populations whose health problems stem from unmet socioeconomic needs, not only is their health impacted by poor housing, a lack of transportation or education, underserviced neighborhoods (eg, food deserts), or strenuous jobs, but they may also lack the means to adopt and record daily health behaviors predetermined by insurers [10]. For example, a person living in an underserviced neighborhood is less likely to have access to adequate sports facilities and, therefore, is less likely to be physically active enough to achieve the performance goals required to stay healthy as determined by insurers [10,11]. The same is true for a single parent who does not have access to childcare services. In addition, a person suffering from depression will find it difficult to be physically active or to prepare and eat healthy meals [12]. People in financially precarious situations also have greater difficulty buying nutritious and healthy food and having access to alternative food systems (eg, food swamps) [13,14]. Thus, vulnerable populations could face a double disadvantage if they are subjected to the PAYL model: They will face more expensive insurance policies as a result of systemic barriers that already affect their living conditions and health problems [15,16].

Second, while digital health technologies may potentially have an added value for people who are relatively young, healthy, educated, financially stable, and living in safe and serviced neighborhoods, demonstrating whether the PAYL model improves health outcomes remains challenging. Because individuals subjectively experience their life trajectory, their history, and their environment [17], some may become dependent on technology as they focus on achieving the required health goals, creating a sort of tyranny of self-measurement. Furthermore, individuals with low levels of digital and health literacy may have difficulty managing and interpreting large amounts of data and health information [9]. Such a situation could lead to other problems, including stress, isolation, low self-esteem, deterioration of quality of life, and cognitive overload (“fatigue alert”) [18,19].

### Placing the Burden of Proof on Clients

To prove that they are respecting the terms of their health insurance contracts, clients must demonstrate that they have adopted and followed through on a set of predetermined health behaviors; for example, they must exhibit that they have taken the required daily number of steps, eaten healthily, frequented green areas, or complied with their doctors’ instructions. Taken on by the client, the burden of proof becomes problematic, especially when the basis for the insurance algorithm’s determinations of the client’s instructions and personalized objectives are unknown or ambiguous. Moreover, the insurer can interpret data in different ways. Indeed, it is difficult to know which criteria and parameters are retained by the insurer to estimate risks and calculate the insurance premium.

As an illustration, a person who trains regularly will have optimal physical health, at least theoretically. However, some physical activities are associated with a higher risk of accidents and injuries (eg, muscle and joint injuries, ligament rupture, osteoarthritis) [20-22]. How will the algorithm distinguish risk-avoidance behavior from risk-taking behavior in this case? Using user-generated data to neutralize risks, especially as certain physical damages become predictable, offers a major advantage to the insurer: both rewardable risk-avoidance behavior and risk-taking behavior may remain profitable for the insurer. Payment based on personal risks would, in the majority of cases, result in the client incurring all or a large part of the costs in the event of damage.

### Extracting Value From Client Data

This issue of carrying the burden of proof also raises the question of how the value generated by the client’s data will be shared. Data collection is a two-way process that involves continuous interaction between the client and the insurer [5,15]. Since data are collected on the basis of the individual’s activities and behaviors, the client is a central contributor to the creation of value for the insurer. Thus, the generated value should be shared between the client and the insurer.

Client-generated data under a PAYL insurance contract could also be used for other purposes, whether or not the client has consented to these other purposes. On this point, regulatory requirements may vary and have different effects depending on the jurisdiction. Health data can be sold or outsourced to data brokers who specialize in selling medical forecasts to other third parties, such as employers, life insurers, and credit companies. Because the information asymmetry currently favors insurers, several data collection issues arise [5,15]. These include, for example, conformity with the country’s sociopolitical norms and regulations; compliance with the principles of freedom and equal rights (eg, possibility to request the deletion of data, or the “right to be forgotten”); client consent to all data collection; client knowledge of data collected by insurers; and data use (or re-use) transparency.

### Mutualization Versus Individualization of Risk

As a social value [23], the promotion of digital health can no longer be examined outside of the social contract that reflects every society’s values [24]. With the PAYL model, publicly funded and universal health systems could gradually migrate from a model of collective solidarity, where risks are mutualized and where citizens receive services corresponding to their needs rather than their ability to pay, to a transactional model, based on the calculation of individual risks. Indeed, the high visibility enabled by this model when calculating individual risks will most likely impact the values of solidarity that characterize collective insurance models [5,15]. It can be hypothesized that not only will the most vulnerable groups be disproportionally affected, but the very basis upon which collective insurance models work will also be eroded.

## Guiding Health Insurance Models Toward Responsible Innovation

The PAYL insurance model can be seen as an innovation, but one that has many pitfalls. To help guide the development of health insurance models that both leverage digital health technologies and meet population health needs, the Responsible Innovation in Health (RIH) framework developed by Silva et al [25] offers an interesting starting point. To our knowledge, the RIH framework is one of the few frameworks that go beyond innovation ethics and includes, in an integrated manner, organizational, environmental, health system, and population health aspects. Furthermore, it emphasizes a collective approach that can shed light on various private, public, and mixed health insurance models while keeping the effectiveness and safety of health innovation at the center of stakeholders’ reflections and actions. This framework aims to support equitable and sustainable health care by fostering the development of innovations along 5 value domains that include 9 attributes [25,26]. Table 1 illustrates how the RIH value domains and attributes can be applied to set responsibility objectives for digitalized health insurance models.

The RIH framework invites those who develop insurance models to respond to the most pressing population needs while reducing health inequalities. To this end, stakeholders who have knowledge about and power over various determinants of health could be involved in identifying and defining a broader digital health dataset, one that makes explicit the systemic facilitators and barriers that affect how people live. This could provide a fairer and more valid representation of living conditions and, eventually, support personalized priorities and objectives according to the contextual factors affecting individual health behaviors. To better address health system needs, insurance algorithms could focus on helping clients manage chronic diseases or comorbidities that put a strain on health systems. An optimized, affordable, and easy-to-use platform or mobile application could be customizable according to clients’ data plans (which can be very expensive in North America) and integrate digital and health literacy functionalities to enable clients to make the most out of their health data. A business model that provides value to both clients and society could share, following a clear data stewardship model, the collected health data with population health researchers who study, for instance, the impact of environmental factors on diabetes, heart disease, sleep disorders, etc. Finally, energy-intensive server farms could run on clean energy to reduce smog levels as well as the incidence of respiratory diseases associated with air pollution. In other words, the RIH framework offers a lens to reconsider how the mutualization of risks may spur innovative insurance models in which benefits may also be mutualized more broadly.

**Table 1:** The application of RIH value domains, attributes, and responsibility objectives for digitalized health insurance models.

Value domain and attribute		Objective
**Population health value**		
	Health relevance	Respond to the collective needs of the population.
	Ethical, legal, and social issues	Mitigate the ethical, legal, and social issues related to health data privacy and security.
	Health inequalities	Contribute to reducing inequalities in access to health care and services (eg, digital and health literacy).
**Health system value**		
	Inclusiveness	Work with stakeholders (eg, clients, health care providers, technology providers) throughout the innovation lifecycle.
	Responsiveness	Appropriately respond to important health system needs (eg, service delivery gaps or demographic changes that call for alternative insurance packages).
	Level and intensity of care	Examine at what level in the system appropriate care can be delivered safely and effectively.
**Economic value**		
	Frugality	Provide an optimized, affordable, and easy-to-use solution to all clients.
**Organizational value**		
	Business model	Follow a business model that provides value to both clients and society.
**Environmental value**		
	Eco-responsibility	Reduce the environmental footprint caused by the production and use of digital technology and the development and use of digital infrastructures and algorithms (eg, servers).

More specifically, if we take the example of clients with low income and with type 2 diabetes, a responsible digitalized health insurance model could capture secure and private data on their living conditions (eg, type of work, types of services and amenities available in their neighborhoods, time constraints related to childcare duties, weekly budget), comorbidities (eg, retinopathy), and digital and health literacy (eg, mother tongue, reading and comprehension levels, knowledge on their disease). It could improve access to mobile technologies and data plans and appropriately secure clients’ consent to share their anonymized health data for health research on type 2 diabetes. In this way, personalized health objectives and behaviors would take into consideration all diabetic clients’ contextual facilitators and barriers. Furthermore, an application could help clients find weekly sales on fresh fruits and vegetables in their area, suggest a safe walking route to the store (if possible), recommend an easy recipe to cook products on sale, and provide a bit of information on how the purchased products help to keep them healthy. As such, rather than focusing on risk-taking or avoidance, healthy behaviors would be contextually scaffolded, and the insurance premium would reward the capturing and sharing of data for research purposes as well as a reduction in the level and intensity of care (eg, reduction in hospitalization rates).

Of course, to ensure the success of such a model, individuals must be willing to share a multitude of data that can provide a holistic picture of their context and needs. This is a major challenge, particularly given the lack of public confidence in how the data will be used in the future, especially around issues of confidentiality, privacy, commercial use, and discrimination [27]. It is essential to have transparent data governance structures and models to ensure the responsible and accountable use of the data for the benefit of the society. In order to increase and maintain confidence, it is also important to go beyond strict legal compliance and ensure real public involvement in data governance through information, transparency, and control [27].

## Conclusions

With the PAYL model, health insurers seek to leverage new digital technologies and value extraction models while adapting to their clients’ shifting health needs. However, as the latter increasingly includes diversified populations whose health is conditioned by broader determinants over which they have little, if any, control, this paper aims to shed light on problematic assumptions and the modus operandi behind this emerging model: the quantification of complex behaviors; shifting the burden of proof to clients who are, in a sense, presumed guilty unless digital trackers can show otherwise; the lack of transparency in how economic value is extracted from client data; and how such models undermine the very principle which, several decades ago, made health insurance a responsible public policy innovation—the mutualization of risk.

At the same time, the PAYL model remains an innovative approach that could help health systems become more efficient and equitable, particularly by supporting the development of healthy habits and adherence to care treatments. Yet, to achieve these objectives, we must consider the complexity inherent in the lives of individuals and communities, as well as the principles that define the social contract of our society, which includes the protection of vulnerable populations. In this respect, the RIH framework could be a major lever to guide the development of responsible digitalized health insurance models that adequately respond to and support the mission of health systems.

In conclusion, by analyzing the PAYL insurance model as a health innovation, this paper contributes to the scholarship by comprehensively applying the RIH framework to explore responsibility features that go beyond innovation ethics. In doing so, this paper also highlights the way in which an alternative digitalized health insurance model could be further developed by public or private institutions, thus providing a knowledge base for future studies.

## Abbreviations

PAYL: Pay-As-You-Live
RIH: Responsible Innovation in Health
